# Embryonic *IGF2* Expression Is Not Associated with Offspring Size among Populations of a Placental Fish

**DOI:** 10.1371/journal.pone.0045463

**Published:** 2012-09-19

**Authors:** Matthew Schrader, Joseph Travis

**Affiliations:** 1 Department of Animal Biology, University of Illinois, Urbana, Illinois, United States of America; 2 Department of Biological Science, Florida State University, Tallahassee, Florida, United States of America; California State University Fullerton, United States of America

## Abstract

In organisms that provision young between fertilization and birth, mothers and their developing embryos are expected to be in conflict over embryonic growth. In mammalian embryos, the expression of *Insulin-like growth factor II* (*IGF2*) plays a key role in maternal-fetal interactions and is thought to be a focus of maternal-fetal conflict. Recent studies have suggested that *IGF2* is also a focus of maternal-fetal conflict in placental fish in the family Poeciliidae. However, whether the expression of *IGF2* influences offspring size, the trait over which mothers and embryos are likely to be in conflict, has not been assessed in a poeciliid. We tested whether embryonic *IGF2* expression varied among four populations of a placental poeciliid that display large and consistent differences in offspring size at birth. We found that *IGF2* expression varied significantly among embryonic stages with expression being 50% higher in early stage embryos than late stage embryos. There were no significant differences among populations in *IGF2* expression; small differences in expression between population pairs with different offspring sizes were comparable in magnitude to those between population pairs with the same offspring sizes. Our results indicate that variation in *IGF2* transcript abundance does not contribute to differences in offspring size among *H. formosa* populations.

## Introduction

In organisms that provision young after fertilization parents and offspring are likely to be in conflict over the level of parental investment with the optimal level of parental investment being higher from the offspring’s perspective than from the parent’s [1]. This conflict is predicted to be an important influence on the evolution of parent-offspring interactions in species with post-natal parental care as well as species such as placental mammals in which mothers provision embryos between fertilization and birth [1], [2], [3], [4]. In placental species, maternal-fetal conflict is hypothesized to influence the evolution of reproductive isolation, epigenetic phenomena such as genomic imprinting, and possibly the evolution of placentation itself from less elaborate forms of viviparity [2], [3], [5], [6].

In species with post-natal parental care, parent-offspring conflict involves behavioral interactions between parents and their dependent young [1], [7], [8]. In contrast, conflict between mothers and their developing embryos is likely to involve the expression of growth enhancing and growth suppressing genes by embryos, as well as the response of mothers to the expression of these genes. Perhaps the most dramatic example of maternal-fetal conflict over maternal investment involves the expression of *Insulin-like growth factor II* (*IGF2*) and the *Insulin-like growth factor II receptor* (*IGF2r*) in mouse embryos [6], [9]. *IGF2* is a potent growth factor while *IGF2r* inhibits prenatal growth by degrading *IGF2*. These two genes are oppositely imprinted in mice with the paternally inherited copy of the growth-enhancing gene (*IGF2*) and the maternally inherited copy of the growth-suppressing gene (*IGF2r*) being expressed in developing embryos [10], [11]. The pattern of imprinting and the phenotypic effects of these two genes are consistent with their involvement in intragenomic conflict over prenatal maternal investment [6].

While most studies of maternal-fetal conflict have focused on mammals, maternal-fetal conflict may also be an important force in placental fish in the family Poeciliidae [12], [13], [14]. With the exception of a single species, all poeciliid fish give birth to fully developed, independent young [15], [16]. However, there is considerable variation among species in both the presence and degree of post-fertilization maternal investment [17]. Some species do not provide developing embryos with nutrients beyond those provided in the egg (lecithotrophs). Other species provision developing embryos via a placenta composed of the maternal ovarian follicle and the embryo’s pericardial sac (matrotrophs). In addition to variation in the presence and degree of matrotrophy, there is variation among populations of some species in the level of matrotrophy [14], [18], [19]. Finally, the mating systems of many poeciliids are characterized by high levels of multiple paternity which is expected to increase the magnitude of maternal-fetal conflict [20].

Two previous studies have examined the role that *IGF2* plays in maternal-fetal conflict in poeciliids. In the first study, Lawton et al. [21] tested whether *IGF2* is imprinted in two matrotrophic poeciliids (*Heterandria formosa* and *Poeciliopsis prolifica*). In both species, *IGF2* was biallelically expressed, suggesting that maternal-fetal conflict has not driven the evolution of genomic imprinting in poeciliids. In the second study, O’Neill et al. [22] found that *IGF2* expression in *H. formosa* is localized in the embryonic contribution to the placenta (the pericardial sac) and that *IGF2* has evolved under strong positive selection in poeciliids. The evidence for positive selection was especially strong in lineages that have evolved placentas recently. These observations are consistent with the hypothesis that *IGF2* has been a focus of maternal-fetal conflict in poeciliid fish.

Studies of the expression and evolution of *IGF2* in poeciliids and its growth enhancing function in mammalian embryos suggest that altering the expression of this gene may allow poeciliid embryos to influence their own growth. While there is ample evidence that *IGF2* expression influences offspring size in mammals [11], no studies have investigated whether embryonic *IGF2* expression influences offspring size at birth in poeciliids. Here we address this gap by testing whether there is an association between embryonic *IGF2* expression and offspring size at birth in four populations of a matrotrophic poeciliid that exhibit large and consistent differences in size at birth.

## Methods

Ethics statement: All work was approved by the Florida State University Institutional Animal Care and Use Committee (protocol number 9321) and carried out in strict accordance with national guidelines. Females were euthanized with an overdose of anesthetic (MS222).

*Heterandria formosa* is a highly matrotrophic poeciliid fish distributed along the southeastern coastal plain of the United States. In addition to being highly matrotrophic, *H. formosa* females simultaneously provision several broods of developing embryos, a phenomenon referred to as superfetation. The combination of matrotrophy and superfetation in this species increases the potential for maternal-fetal conflict and sibling competition for maternally supplied resources [14], [23]. In addition, previous work has demonstrated that variation among populations in offspring size at birth is due to a combination of maternal and direct effects of offspring genotype on offspring size [14], [19].

We quantified *IGF2* expression in embryos from four *H. formosa* populations located in North Florida: Moore Lake (ML), Trout Pond (TP), Wacissa River (WR), and Wakulla Springs (WS). Offspring from Wacissa River and Wakulla Springs are, on average 40% larger than offspring from Moore Lake and Trout Pond. These differences are present under field and laboratory conditions and have been the focus of study for over 20 years [14], [19], [24], [25], [26], [27]. In two previous studies involving these populations, we tested whether differences in offspring size at birth are due to differences in the size of mature ova or differences in post-fertilization maternal investment. These studies show that differences between populations in offspring size at birth are reflected in the size of early stage embryos and are due to differences in post-fertilization maternal provisioning not differences in the size of mature ova [14], [19].

We collected pregnant females from each population during the first week of May 2011. Females were euthanized with an overdose of anesthetic (MS222) and dissected in the field to remove the ovary containing developing embryos. The ovary of each female was preserved in RNAlater (Qiagen, Valencia, CA) and placed on ice until samples were retuned to the lab and stored at -20 C. We dissected each preserved ovary to remove developing embryos. Embryos were placed into a developmental stage following Travis et al. [26]. We retained embryos from stages 2-5 (early-eyed, mid-eyed, late-eyed, and very late-eyed embryos) and placed broods from each stage of each female in a new tube containing RNAlater.

We quantified the expression of *IGF2* in embryos relative to a control gene, *Elongation factor 1 α* (*EF1 α*) using quantitative real time RTPCR (QPCR). This gene is frequently used as a control gene in QPCR studies (e.g. [28]) and preliminary work indicated that its expression varied little among embryonic stages and populations. Whole embryos were homogenized using QIAshredders (Qiagen, Valencia, CA) and RNA was extracted from whole embryo homogenates using RNeasy mini kits (Qiagen, Valencia, CA). Total RNA was reverse transcribed into cDNA using Super Script III, First Stand Synthesis Supermix (Invitrogen, Carlsbad, CA) according to the manufacturer’s instructions.

*IGF2* and *EF1α* primer sequences were taken from [21], [28]. QPCR reactions contained 5 µl SYBR® Green PCR master mix, 1 µl of 2.5 µM forward primer, 1 µl of 2.5 µM reverse primer, and 3 µl cDNA. Three technical replicates of each cDNA sample were performed and control reactions with no cDNA template were included on each plate to determine the level of background contamination. A 5-step serial dilution standard curve was generated for each gene using pooled cDNA from embryos from each developmental stage. Quantification of gene expression was performed with an ABI Prism 7900 sequence detector (Applied Biosystems, Foster City, CA). We estimated the efficiency of the PCR reaction for each gene (E) using the slope of CT on log quantity from the dilution series. Relative *IGF2* expression (RE) was calculated as:
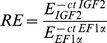



In total, we measured *IGF2* expression from 203 embryos from 34 females. While the total numbers of embryos were comparable among ML, TP, WR, and WS (46, 51, 48, and 58 embryos respectively), the numbers of female parents were more variable (6, 10, 8, and 10 respectively). We used between 7 and 22 embryos at each combination of population and stage except stage 2 at ML, for which we obtained only 2 embryos.

To analyze the expression data, we performed a two-way ANOVA, considering stage and population as fixed effects and pooling variance attributable to the identity of individual mothers (which was not statistically significant) into the residual variation. We report these results; however, we performed several alternative analyses with different statistical assumptions that yielded nearly identical results. We performed all analyses on log-transformed relative expression levels so that the assumptions of linear analyses would be met and used Type III SS to assess statistical significance.

## Results

**Figure 1:**
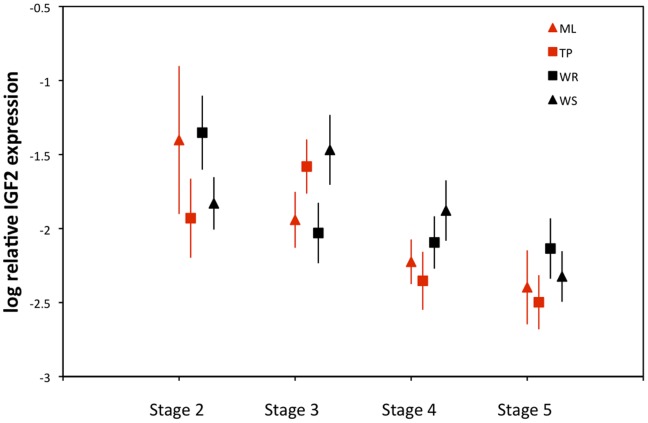
Log relative *IGF2* expression (means ± SE) in stage 2, 3, 4 and 5 *H. formosa* embryos from Moore Lake (ML, red triangles), Trout Pond (TP, red squares), Wacissa River (WR, black squares), and Wakulla Springs (WS, black triangles).

Levels of *IGF2* expression were highly variable, even among embryos of the same female at the same stage. Nonetheless, there was a regular decrease in the average levels of *IGF2* expression from the earliest to the latest embryonic stages (Fig. 1); on the logarithmic scale, average expression in stage-5 embryos was about 50% lower than that for stage-2 embryos. The variation among developmental stages was significant (two-way ANOVA, F_3, 195_ = 10.44, P<0.0001); post-hoc pairwise comparisons using Tukey’s method found that the averages fell into two groups, one including stages 2 and 3 and another including stages 4 and 5. These results are consistent with those of Lawton et al. [21] who found *IGF2* expression to peak in early and mid-eyed *H. formosa* embryos.

**Figure 2:**
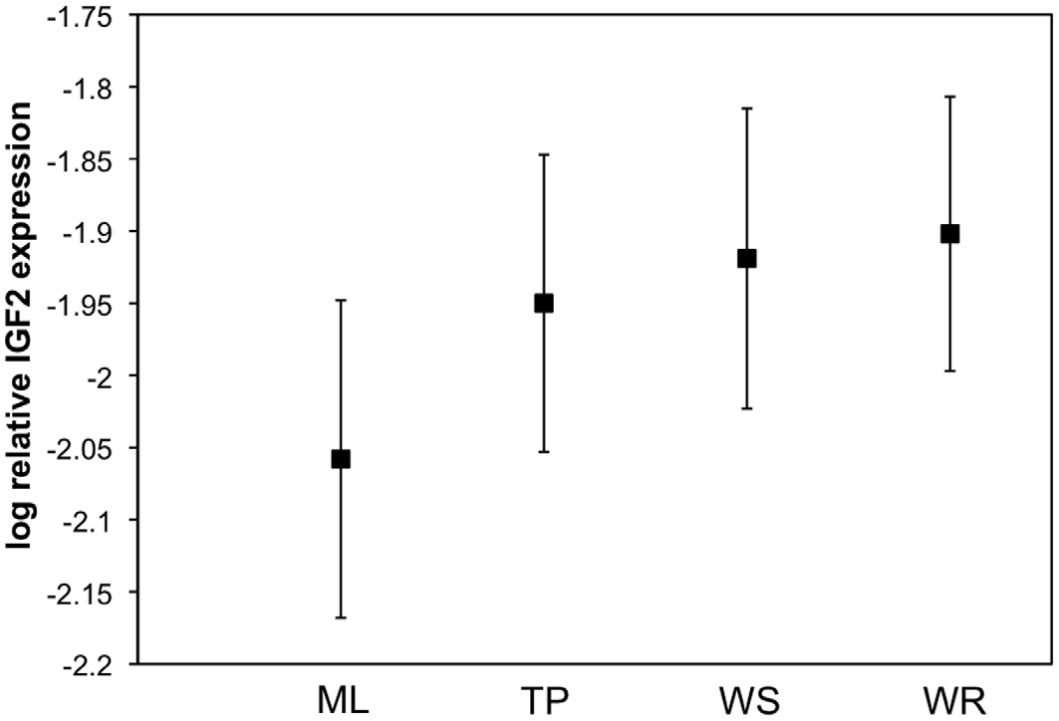
Log relative *IGF2* expression (least squares mean ± SE) in *H. formosa* embryos from Moore Lake (ML), Trout Pond (TP), Wacissa River (WR), and Wakulla Springs (WS). Values are averaged across developmental stages.

Despite the fact that offspring size varies dramatically among these populations, there was no evidence for variation among their average levels of *IGF2* expression (Fig. 2). On the logarithmic scale, there was only an 8% difference in relative *IGF2* expression between the populations with the lowest and highest average expression levels (ML and WS respectively). There were no significant differences among populations and less than 1% of the total variance in *IGF2* expression was attributable to population (Two-way ANOVA; F_3, 195_ = 0.43, P = 0.73).

## Discussion

*IGF2* is a major axis of maternal-fetal conflict in mammals and is hypothesized to be a focus of maternal-fetal conflict in poeciliid fish. The evidence that maternal-fetal conflict in mammals involves *IGF2* is especially compelling for two reasons. First, *IGF2* expression has a clear influence on offspring size at birth, the trait over which mothers and embryos are expected to be in conflict [11]. Second, *IGF2* is imprinted with the paternal copy expressed in developing embryos. This pattern of expression is consistent with the kinship theory of genomic imprinting [6]. The evidence for maternal-fetal conflict over *IGF2* expression in poeciliids is based mainly on the observation that it is expressed in the poeciliid placenta and has evolved under strong positive selection, especially in matrotrophic lineages [22]. However, *IGF2* is not imprinted in placental poeciliids [21] and our results indicate that divergence in offspring size at birth does not involve divergence in *IGF2* expression.

The absence of an association between *IGF2* expression and offspring size in our study is surprising, considering that *IGF2* expression has been shown to differ between fast and slow growing strains of some fish species [29]. This suggests that the expression of other growth factors (e.g. *IGF1*) may be an important determinant of offspring size in *H. formosa*. Alternatively, offspring size may be influenced by the interaction between *IGF2* expression and the expression of other genes. For example, O’Neill et al. [22] found that the two *IGF2* sites with the strongest signature of positive selection in matrotrophic poeciliids were adjacent to the type 1 *IGF* receptor. It is possible that the interaction between the expression of *IGF2* and the *IGF1* receptor in *H. formosa* embryos influences offspring size at birth.

One might argue that our results are not definitive because even very small differences in *IGF2* expression, smaller than those we had the statistical power to detect, could induce substantial differences in offspring size. Indeed, studies of mice suggest that small changes in *IGF2* expression may influence offspring size. For example, a targeted mutation in *IGF2* resulting in a 10% decline in *IGF2* expression was associated with a 60% decline in offspring size in mice [9], [11]. It is unlikely that this is the case in our data. The difference in *IGF2* expression between pairs of populations characterized by small and large offspring was between 8% (Moore Lake and Wakulla Springs) and about 1.6% (Trout Pond and Wacissa River). This range is comparable to the differences seen between pairs with similarly sized offspring, 6% between Moore Lake and Trout Pond and 0.9% between Wacissa River and Wakulla Springs (Figure 2). This pattern argues that divergence in offspring size among these populations does not involve the regulation of this gene.

The rapid evolution of *IGF2* in poeciliid fish is suggests that this gene plays a major role in the evolution of placentation. However, if *IGF2* expression is a focal point of maternal-fetal conflict in placental poeciliids, our observations suggest this conflict is not manifested at the level of *IGF2* mRNA abundance in *H. formosa.* It is possible that post-transcriptional processing of *IGF2* or interactions between *IGF2* and other genes determine the outcome of maternal-fetal interactions in poeciliids. The development of genomic resources for placental poeciliid fish [30] may allow broader transcriptomic comparisons among these populations, which in turn could identify the genes important in maternal-fetal conflict and the elaboration of matrotrophy.
